# Routine use of microarray-based gene expression profiling to identify patients with low cytogenetic risk acute myeloid leukemia: accurate results can be obtained even with suboptimal samples

**DOI:** 10.1186/1755-8794-5-6

**Published:** 2012-01-30

**Authors:** Diane Raingeard de la Blétière, Odile Blanchet, Pascale Cornillet-Lefèbvre, Anne Coutolleau, Laurence Baranger, Franck Geneviève, Isabelle Luquet, Mathilde Hunault-Berger, Annaelle Beucher, Aline Schmidt-Tanguy, Marc Zandecki, Yves Delneste, Norbert Ifrah, Philippe Guardiola

**Affiliations:** 1 Plateforme SNP, Transcriptome & Epigénomique, Centre Hospitalier Universitaire, Angers, France; 2 Institut National de la Santé et de la Recherche Médicale, Unité 892, Centre de Recherche sur le Cancer Nantes Angers et UMR_S 892, Université d'Angers, Angers, France; 3 Laboratoire d'Hématologie, Centre Hospitalier Universitaire, Angers, France; 4 Laboratoire d'Hématologie, Centre Hospitalier Universitaire, Reims, France; 5 Laboratoire de Génétique, Centre Hospitalier Universitaire, Angers, France; 6 Service des Maladies du Sang, Centre Hospitalier Universitaire, Angers, France

## Abstract

**Background:**

Gene expression profiling has shown its ability to identify with high accuracy low cytogenetic risk acute myeloid leukemia such as acute promyelocytic leukemia and leukemias with t(8;21) or inv(16). The aim of this gene expression profiling study was to evaluate to what extent suboptimal samples with low leukemic blast load (range, 2-59%) and/or poor quality control criteria could also be correctly identified.

**Methods:**

Specific signatures were first defined so that all 71 acute promyelocytic leukemia, leukemia with t(8;21) or inv(16)-AML as well as cytogenetically normal acute myeloid leukemia samples with at least 60% blasts and good quality control criteria were correctly classified (training set). The classifiers were then evaluated for their ability to assign to the expected class 111 samples considered as suboptimal because of a low leukemic blast load (n = 101) and/or poor quality control criteria (n = 10) (test set).

**Results:**

With 10-marker classifiers, all training set samples as well as 97 of the 101 test samples with a low blast load, and all 10 samples with poor quality control criteria were correctly classified. Regarding test set samples, the overall error rate of the class prediction was below 4 percent, even though the leukemic blast load was as low as 2%. Sensitivity, specificity, negative and positive predictive values of the class assignments ranged from 91% to 100%. Of note, for acute promyelocytic leukemia and leukemias with t(8;21) or inv(16), the confidence level of the class assignment was influenced by the leukemic blast load.

**Conclusion:**

Gene expression profiling and a supervised method requiring 10-marker classifiers enable the identification of favorable cytogenetic risk acute myeloid leukemia even when samples contain low leukemic blast loads or display poor quality control criterion.

## Background

Prognostic evaluation is a critical step in newly diagnosed patients with acute myeloid leukemia (AML) in order to identify those at high risk of relapse. For AML patients, cytogenetic abnormalities as well as gene mutations and/or hyper-expressions detected at diagnosis are the main prognostic factors guiding the initial treatment strategy in a risk-oriented manner [1-4]. Hypergranular acute promyelocytic leukemia (APL), as well as AMLs with either translocation t(8;21)(q22;q22) [t(8;21)-AMLs] or inversion inv(16)(p13q22)/t(16;16)(p13;q22) [inv(16)-AMLs], are well-defined entities associated with a favorable outcome [1,3]. They can be distinguished from all other AML subtypes based on specific chromosomal alterations and fusion genes: *PML/RAR-alpha *fusion gene with reciprocal translocation t(15;17)(q24;q21) for APLs, *AML1/ETO *(also called *RUNX1/RUNX1T1*) fusion gene for t(8;21)-AMLs, and *CBFB/MYH11 *fusion gene for AMLs with either inv(16)(q21;q22) or balanced reciprocal translocation t(16;16)(q21;q22). Of note, in up to 15 percent of APLs, no translocation t(15;17)(q24;q21) is detected by conventional cytogenetics at diagnosis, despite *PML/RARA *fusion gene is detected using molecular assays [5,6]. Similarly, cryptic t(8;21)(q22;q22) and inv(16)(q21;q22), undetected by conventional cytogenetics, have also been reported [7-9].

With samples containing a high leukemic blast load, microarray-based gene expression profiling (GEP) and class prediction analyses have demonstrated their ability to assign AML samples to one of these three well characterized favorable cytogenetic risk AML subtypes, with high accuracy and low error rates [10-18]. One of the largest class prediction analyses in AML achieved 100 percent classification accuracy with respect to APL, t(8;21)-AML, and inv(16)-AML subtypes, indeed [10]. However, in the majority of those studies, the minimum percentage of leukemic cells within each sample is most often above 60 percent [10-18], an arbitrary threshold that is significantly different from the one used by cytologists for the diagnosis of AML, i.e., excess of blasts greater or equal to 20 percent.

To our knowledge, the impact of the leukemic blast load and/or of the sample quality on microarray-derived class prediction results has not been specifically studied in AMLs. For centers wishing to integrate microarray-based GEP in a routine prognostic workflow for newly diagnosed AML patients, one critical issue is the ability to perform accurate class prediction analysis with suboptimal samples, i.e., containing as low as 20 percent blasts and even below, and/or not fulfilling all quality control criteria along their process.

In this study, GEP was first used to define a limited set of markers allowing to correctly classify 71 patients with either APL, t(8;21)-AML, inv(16)-AML or cytogenetically normal AML (NK-AML) based on samples containing at least 60 percent of leukemic blasts and characterized by good quality control criteria (training set including optimal samples). The classifiers derived from this first supervised analysis were then evaluated for their ability to assign to the correct class 111 suboptimal samples with low leukemic blast load (from 2 to 59 percent) and/or poor quality control criteria (test set including suboptimal samples), as well as duplicates of three AML cell lines.

## Methods

### Characteristics of the patients and samples

A total of 182 bone marrow or peripheral blood samples from 97 AML patients, diagnosed with either APL (n = 18), t(8;21)-AML (n = 19), inv(16)-AML (n = 29), or NK-AML (n = 31), followed at Angers University Hospital (n = 72) or Reims University Hospital (n = 25), were analyzed, as well as duplicated samples of NB4, Kasumi-1, and ME-1 cell lines (n = 6 samples), which are derived from patients with APL, t(8;21)-AML and inv(16)-AML, respectively. In addition, 18 samples of unique (n = 9) or pooled (n = 9) normal bone marrows obtained from 12 healthy volunteers were included in the study. The main characteristics of AML patients and samples are summarized in Tables 1 and 2. Conventional cytogenetic banding, fluorescence *in situ *hybridization, and RT-PCR analysis of fusion gene transcripts were used to identify patients with APL, t(8;21)-AML or inv(16)-AML, as previously reported [19]. Analysis of *NPM1, FLT3 and CEBPA *for mutations was also performed for all patients [20-23]. All participants gave their written informed consent, and the study was approved by the Ethical Committee of Angers University Hospital.

**Table 1:** Characteristics of the patients.

Covariates	Overall	APLs	t(8;21)-AMLs	inv(16)-AMLs	NK-AMLs
Patients	N = 97	N = 18	N = 19	N = 29	N = 31
Gender					
Males	44	7	13	15	9
Females	53	11	6	14	22
Age at diagnosis					
Median (years)	54	56	53	38	60
Range	18-87	19-87	18-84	18-70	25-78
Leukocytosis at diagnosis					
WBC ≥ 30 G/L	39	2	6	12	19
FAB classification					
M1 or M2	45	0	18	6	21
M3	18	18	0	0	0
M4 or M5	34	0	0	24	10
Cytogenetics					
Expected anomaly*	62	16	19	27	0
Normal karyotype	34	2	0	1	31
Karyotype failure	1	0	0	1	0
Gene mutations					
FLT3-ITD^$^	19	2	1	2	14
FLT3-D835^&^	7	2	2	2	1
NPM1	18	0	0	1	17
CEBPA (mono-allelic)	4	0	2	1	1

WBC, white blood cells. * t(15;17), t(8;21), inv(16) or t(16;16) for APL, t(8;21)-AML and inv(16)-AML, respectively detected. ^$ ^Internal tandem duplication. ^&^Mutation Asp835 of the tyrosine kinase domain.

**Table 2:** Characteristics of the samples.

Covariates	Overall	APLs	t(8;21)- AMLs	inv(16)-AMLs	NK-AMLs
Samples*	N = 206	N = 40	N = 39	N = 52	N = 57
Groups					
Training Set AML samples	71	14	14	15	28
Training Set NBM^&^samples	18	--	--	--	--
Test Set AML samples*	111	24	23	35	29
Test Set AML cell lines	6	2	2	2	--
Samples with optimal QCC^#^					
Blast % < 60% (undiluted)	22	3	5	11	3
Diluted at 50%	28	5	5	5	13
Diluted at 75%	27	5	4	5	13
Diluted at 90%	12	4	4	4	0
Diluted at 95%	12	4	4	4	0
Overall, blasts < 60%	101	21	22	29	29
Overall, blasts ≥ 40% < 60%	23	2	2	6	13
Overall, blasts ≥ 20% < 40%	40	6	9	10	15
Overall, blasts ≥ 10% < 20%	15	4	4	6	1
Overall, blasts ≥ 5% < 10%	14	5	4	5	0
Overall, blasts < 5%	9	4	3	2	0
Samples with poor QCC^#^	10	3	1	6	0
cRNA ^$ ^< 750 ng	7	1	1	5	0
Low RIN^%^	2	2	0	0	0
Low RIN + low cRNA	1	0	0	1	0

* including the three AML cell lines. ^# ^quality control criteria. ^$ ^labeled cRNA to be hybridized on Illumina Beadchips.

^% ^RNA integrity number lower than 7.00. ^&^NBM, normal bone marrow.

The gene expression dataset, generated from 206 samples (182 AML samples, duplicated samples of NB4, Kasumi-1, and ME-1 cell lines, 18 samples of unique or combined normal bone marrows), was divided into a Training Set (n = 89 samples) and a Test Set (n = 117 samples). The Training Set, used to identify specific class markers (classifiers), included 18 samples of unique or pooled normal bone marrows (see details about the pools below) as well as 71 AML samples obtained from patients (bone marrow samples, n = 48; peripheral blood samples, n = 23) newly diagnosed with APL (n = 14 patients), t(8;21)-AML (n = 14 patients), inv(16)-AML (n = 15 patients) or NK-AML (n = 28 patients), which contained at least 60 percent blasts. The class prediction analysis was performed on a Test Set including 111 suboptimal AML samples (bone marrow samples, n = 78; peripheral blood samples, n = 33 obtained from 53 patients), i.e., with a low leukemic blast load (n = 101 samples containing from 2 to 56 percent blasts) and/or poor quality control criteria (n = 10 samples) as well as duplicated samples of NB4, Kasumi-1, and ME-1 cell lines (n = 6 samples). Among test samples with optimal quality control criteria, 22 originally contained less than 60% blasts (range, 5-56 percent blasts), whereas 79 were high blast load ones artificially diluted within one of the five pools of normal bone marrows (see details below and Tables 2 and 3). The 10 samples with suboptimal quality control criteria were characterized by either a low RNA integrity number suggestive of RNA degradation (n = 2), a low cRNA amount obtained after total RNA amplification and labeling (cRNA hybridized on BeadChips < 750 micrograms) (n = 7) or both (n = 1) (Tables 2 and 3).

**Table 3:** Characteristics and class assignment of the 117 Test Set samples.

UPN	Cell Source	Time	Blasts %	Dilution	QCC	Real Class	Assigned Class	Confidence
NB4	BM	Diagnosis	100	No Dilution	OK	APLs Test Set	APLs	10.802
NB4	BM	Diagnosis	100	No Dilution	OK	APLs Test Set	APLs	17.507
UPN53	BM	Diagnosis	43	Dilution 50%	OK	APLs Test Set	APLs	26.028
UPN22	BM	Diagnosis	40	Dilution 50%	OK	APLs Test Set	APLs	29.91
UPN65	BM	Diagnosis	39	Dilution 50%	OK	APLs Test Set	APLs	26.403
UPN40	BM	Diagnosis	33	Dilution 50%	OK	APLs Test Set	APLs	29.91
UPN50	PB	Diagnosis	27	Dilution 50%	OK	APLs Test Set	APLs	19.2
UPN53	BM	Diagnosis	22	Dilution 75%	OK	APLs Test Set	APLs	20.904
UPN22	BM	Diagnosis	20	Dilution 75%	OK	APLs Test Set	APLs	29.91
UPN65	BM	Diagnosis	20	Dilution 75%	OK	APLs Test Set	APLs	15.763
UPN40	BM	Diagnosis	16	Dilution 75%	OK	APLs Test Set	APLs	26.425
UPN5	PB	Diagnosis	15	No Dilution	OK	APLs Test Set	APLs	17.193
UPN50	PB	Diagnosis	14	Dilution 75%	OK	APLs Test Set	APLs	10.46
UPN48	PB	Diagnosis	11	No Dilution	OK	APLs Test Set	APLs	23.005
UPN53	BM	Diagnosis	9	Dilution 90%	OK	APLs Test Set	APLs	16.749
UPN22	BM	Diagnosis	8	Dilution 90%	OK	APLs Test Set	APLs	15.781
UPN40	BM	Diagnosis	7	Dilution 90%	OK	APLs Test Set	APLs	10.143
UPN86	PB	Diagnosis	7	No Dilution	OK	APLs Test Set	APLs	11.641
UPN50	PB	Diagnosis	6	Dilution 90%	OK	APLs Test Set	APLs	2.661
UPN22	BM	Diagnosis	4	Dilution 95%	OK	APLs Test Set	APLs	7.665
UPN53	BM	Diagnosis	4	Dilution 95%	OK	APLs Test Set	APLs	1.201
UPN40	BM	Diagnosis	3	Dilution 95%	OK	APLs Test Set	APLs	10.667
UPN50	PB	Diagnosis	3	Dilution 95%	OK	APLs Test Set	APLs	4.337
UPN59	PB	Diagnosis	70	No Dilution	Low RIN	APLs Test Set	APLs	26.497
UPN82	BM	Diagnosis	64	No Dilution	Low RIN	APLs Test Set	APLs	22.822
UPN13	BM	Diagnosis	30	No Dilution	Low cRNA	APLs Test Set	APLs	18.651
Kasumi-1	BM	Diagnosis	100	No Dilution	OK	t(8;21)-AMLs Test Set	t(8;21)-AMLs	5.184
Kasumi-1	BM	Diagnosis	100	No Dilution	OK	t(8;21)-AMLs Test Set	t(8;21)-AMLs	1.525
UPN30	PB	Diagnosis	49	Dilution 50%	OK	t(8;21)-AMLs Test Set	t(8;21)-AMLs	19.199
UPN69	PB	Diagnosis	41	Dilution 50%	OK	t(8;21)-AMLs Test Set	t(8;21)-AMLs	30.674
UPN51	BM	Relapse	38	Dilution 50%	OK	t(8;21)-AMLs Test Set	t(8;21)-AMLs	20.609
UPN76	PB	Diagnosis	31	No Dilution	OK	t(8;21)-AMLs Test Set	t(8;21)-AMLs	32.067
UPN69	BM	Diagnosis	30	Dilution 50%	OK	t(8;21)-AMLs Test Set	t(8;21)-AMLs	25.183
UPN26	BM	Diagnosis	27	No Dilution	OK	t(8;21)-AMLs Test Set	t(8;21)-AMLs	14.89
UPN30	PB	Diagnosis	25	Dilution 75%	OK	t(8;21)-AMLs Test Set	t(8;21)-AMLs	17.719
UPN61	BM	Diagnosis	24	Dilution 50%	OK	t(8;21)-AMLs Test Set	t(8;21)-AMLs	30.674
UPN69	PB	Diagnosis	21	Dilution 75%	OK	t(8;21)-AMLs Test Set	t(8;21)-AMLs	27.738
UPN21	BM	Diagnosis	21	No Dilution	OK	t(8;21)-AMLs Test Set	t(8;21)-AMLs	24.116
UPN95	BM	Diagnosis	20	No Dilution	OK	t(8;21)-AMLs Test Set	t(8;21)-AMLs	30.674
UPN51	BM	Relapse	19	Dilution 75%	OK	t(8;21)-AMLs Test Set	t(8;21)-AMLs	10.519
UPN94	PB	Diagnosis	16	No Dilution	OK	t(8;21)-AMLs Test Set	t(8;21)-AMLs	28.631
UPN69	BM	Diagnosis	15	Dilution 75%	OK	t(8;21)-AMLs Test Set	t(8;21)-AMLs	22.744
UPN30	PB	Diagnosis	10	Dilution 90%	OK	t(8;21)-AMLs Test Set	t(8;21)-AMLs	16.958
UPN69	PB	Diagnosis	6	Dilution 90%	OK	t(8;21)-AMLs Test Set	t(8;21)-AMLs	13.468
UPN30	PB	Diagnosis	5	Dilution 95%	OK	t(8;21)-AMLs Test Set	t(8;21)-AMLs	13.581
UPN61	BM	Diagnosis	5	Dilution 90%	OK	t(8;21)-AMLs Test Set	t(8;21)-AMLs	28.443
UPN51	BM	Diagnosis	4	Dilution 90%	OK	t(8;21)-AMLs Test Set	t(8;21)-AMLs	3.096
UPN69	PB	Diagnosis	3	Dilution 95%	OK	t(8;21)-AMLs Test Set	t(8;21)-AMLs	12.875
UPN51	BM	Diagnosis	2	Dilution 95%	OK	t(8;21)-AMLs Test Set	t(8;21)-AMLs	4.6
UPN61	BM	Diagnosis	2	Dilution 95%	OK	t(8;21)-AMLs Test Set	t(8;21)-AMLs	19.237
UPN30	PB	Diagnosis	25	No Dilution	Low cRNA	t(8;21)-AMLs Test Set	t(8;21)-AMLs	15.256
ME-1	BM	Diagnosis	100	No Dilution	OK	inv(16)-AMLs Test set	inv(16)-AMLs	15.836
ME-1	BM	Diagnosis	100	No Dilution	OK	inv(16)-AMLs Test set	inv(16)-AMLs	18.901
UPN52	BM	Diagnosis	56	No Dilution	OK	inv(16)-AMLs Test set	inv(16)-AMLs	26.467
UPN45	BM	Diagnosis	50	Dilution 50%	OK	inv(16)-AMLs Test set	inv(16)-AMLs	30.188
UPN31	BM	Diagnosis	50	No Dilution	OK	inv(16)-AMLs Test set	inv(16)-AMLs	29.511
UPN88	BM	Diagnosis	46	No Dilution	OK	inv(16)-AMLs Test set	inv(16)-AMLs	31.576
UPN47	BM	Diagnosis	41	Dilution 50%	OK	inv(16)-AMLs Test set	inv(16)-AMLs	30.517
UPN37	BM	Diagnosis	40	No Dilution	OK	inv(16)-AMLs Test set	inv(16)-AMLs	28.551
UPN9	BM	Diagnosis	35	No Dilution	OK	inv(16)-AMLs Test set	inv(16)-AMLs	31.083
UPN87	PB	Diagnosis	35	No Dilution	OK	inv(16)-AMLs Test set	inv(16)-AMLs	29.511
UPN10	BM	Diagnosis	33	Dilution 50%	OK	inv(16)-AMLs Test set	inv(16)-AMLs	17.561
UPN43	PB	Diagnosis	31	No Dilution	OK	inv(16)-AMLs Test set	inv(16)-AMLs	24.289
UPN52	BM	Diagnosis	28	Dilution 50%	OK	inv(16)-AMLs Test set	inv(16)-AMLs	24.608
UPN45	BM	Diagnosis	25	Dilution 75%	OK	inv(16)-AMLs Test set	inv(16)-AMLs	23.687
UPN67	PB	Diagnosis	24	No Dilution	OK	inv(16)-AMLs Test set	inv(16)-AMLs	31.576
UPN36	PB	Diagnosis	23	No Dilution	OK	inv(16)-AMLs Test set	inv(16)-AMLs	27.432
UPN47	BM	Diagnosis	20	Dilution 75%	OK	inv(16)-AMLs Test set	inv(16)-AMLs	16.733
UPN37	BM	Diagnosis	20	Dilution 50%	OK	inv(16)-AMLs Test set	inv(16)-AMLs	26.644
UPN10	BM	Diagnosis	16	Dilution 75%	OK	inv(16)-AMLs Test set	inv(16)-AMLs	10.252
UPN52	BM	Diagnosis	14	Dilution 75%	OK	inv(16)-AMLs Test set	inv(16)-AMLs	6.543
**UPN37**	**PB**	**Relapse**	**14**	**No Dilution**	**OK**	**inv(16)-AMLs Test set**	**t(8;21)-AMLs**	**4.33**
UPN45	BM	Diagnosis	10	Dilution 90%	OK	inv(16)-AMLs Test set	inv(16)-AMLs	14.568
UPN47	BM	Diagnosis	10	Dilution 90%	OK	inv(16)-AMLs Test set	inv(16)-AMLs	15.298
UPN37	BM	Diagnosis	10	Dilution 75%	OK	inv(16)-AMLs Test set	inv(16)-AMLs	20.423
UPN10	BM	Diagnosis	7	Dilution 90%	OK	inv(16)-AMLs Test set	inv(16)-AMLs	8.479
**UPN52**	**BM**	**Diagnosis**	**6**	**Dilution 90%**	**OK**	**inv(16)-AMLs Test set**	**APLs**	**0.6**
UPN45	BM	Diagnosis	5	Dilution 95%	OK	inv(16)-AMLs Test set	inv(16)-AMLs	15.056
UPN47	BM	Diagnosis	5	Dilution 95%	OK	inv(16)-AMLs Test set	inv(16)-AMLs	2.293
**UPN7**	**BM**	**Relapse**	**5**	**No Dilution**	**OK**	**inv(16)-AMLs Test set**	**t(8;21)-AMLs**	**0.473**
UPN10	BM	Diagnosis	4	Dilution 95%	OK	inv(16)-AMLs Test set	inv(16)-AMLs	3.715
UPN52	BM	Diagnosis	3	Dilution 95%	OK	inv(16)-AMLs Test set	inv(16)-AMLs	3.79
UPN6	BM	Diagnosis	74	No Dilution	Low cRNA	inv(16)-AMLs Test set	inv(16)-AMLs	27.753
UPN45	BM	Diagnosis	25	No Dilution	Low cRNA	inv(16)-AMLs Test set	inv(16)-AMLs	31.005
UPN47	BM	Diagnosis	20	No Dilution	Low cRNA	inv(16)-AMLs Test set	inv(16)-AMLs	26.009
UPN79	BM	Diagnosis	47	No Dilution	Low cRNA	inv(16)-AMLs Test set	inv(16)-AMLs	29.75
UPN90	PB	Diagnosis	9	No Dilution	Low cRNA	inv(16)-AMLs Test set	inv(16)-AMLs	2.693
UPN23	BM	Diagnosis	27	No Dilution	Low RIN + Low cRNA	inv(16)-AMLs Test set	inv(16)-AMLs	24.111
**UPN60**	**PB**	**Diagnosis**	**50**	**No Dilution**	**OK**	**NK-AMLs Test Set**	**inv(16)-AMLs**	**0.948**
UPN72	BM	Diagnosis	49	Dilution 50%	OK	NK-AMLs Test Set	NK-AMLs	26.707
UPN8	BM	Diagnosis	48	Dilution 50%	OK	NK-AMLs Test Set	NK-AMLs	27.354
UPN44	PB	Diagnosis	47	Dilution 50%	OK	NK-AMLs Test Set	NK-AMLs	28.541
UPN73	PB	Diagnosis	47	Dilution 50%	OK	NK-AMLs Test Set	NK-AMLs	19.624
UPN97	BM	Diagnosis	47	Dilution 50%	OK	NK-AMLs Test Set	NK-AMLs	22.934
UPN1	PB	Diagnosis	46	Dilution 50%	OK	NK-AMLs Test Set	NK-AMLs	26.707
UPN66	BM	Diagnosis	46	Dilution 50%	OK	NK-AMLs Test Set	NK-AMLs	23.454
UPN78	PB	Diagnosis	46	Dilution 50%	OK	NK-AMLs Test Set	NK-AMLs	28.541
UPN25	BM	Diagnosis	43	Dilution 50%	OK	NK-AMLs Test Set	NK-AMLs	21.699
UPN38	BM	Diagnosis	40	Dilution 50%	OK	NK-AMLs Test Set	NK-AMLs	26.707
UPN74	BM	Diagnosis	40	Dilution 50%	OK	NK-AMLs Test Set	NK-AMLs	19.777
UPN89	BM	Diagnosis	40	Dilution 50%	OK	NK-AMLs Test Set	NK-AMLs	29.115
UPN19	BM	Diagnosis	39	No Dilution	OK	NK-AMLs Test Set	NK-AMLs	25.313
UPN11	BM	Diagnosis	37	Dilution 50%	OK	NK-AMLs Test Set	NK-AMLs	29.776
UPN96	PB	Diagnosis	35	No Dilution	OK	NK-AMLs Test Set	NK-AMLs	27.303
UPN72	BM	Diagnosis	25	Dilution 75%	OK	NK-AMLs Test Set	NK-AMLs	26.707
UPN8	BM	Diagnosis	24	Dilution 75%	OK	NK-AMLs Test Set	NK-AMLs	27.357
UPN44	PB	Diagnosis	24	Dilution 75%	OK	NK-AMLs Test Set	NK-AMLs	29.235
UPN97	BM	Diagnosis	24	Dilution 75%	OK	NK-AMLs Test Set	NK-AMLs	24.035
UPN1	PB	Diagnosis	23	Dilution 75%	OK	NK-AMLs Test Set	NK-AMLs	26.707
UPN66	BM	Diagnosis	23	Dilution 75%	OK	NK-AMLs Test Set	NK-AMLs	19
UPN73	PB	Diagnosis	23	Dilution 75%	OK	NK-AMLs Test Set	NK-AMLs	24.104
UPN78	PB	Diagnosis	23	Dilution 75%	OK	NK-AMLs Test Set	NK-AMLs	28.541
UPN25	BM	Diagnosis	21	Dilution 75%	OK	NK-AMLs Test Set	NK-AMLs	18.99
UPN38	BM	Diagnosis	20	Dilution 75%	OK	NK-AMLs Test Set	NK-AMLs	25.107
UPN74	BM	Diagnosis	20	Dilution 75%	OK	NK-AMLs Test Set	NK-AMLs	19.705
UPN89	BM	Diagnosis	20	Dilution 75%	OK	NK-AMLs Test Set	NK-AMLs	26.555
UPN11	BM	Diagnosis	19	Dilution 75%	OK	NK-AMLs Test Set	NK-AMLs	24.587

UPN, unique patient number. BM, bone marrow. PB, peripheral blood. QCC, quality control criteria.

Low cRNA, amount of labeled cRNA hybridized on the BeadChip below 750 ng. Low RIN, RNA integrity number below 7.00.

### Sample preparation for gene expression profiling

Blasts and mononuclear cells were purified by Ficoll-Hypaque density gradient centrifugation from bone marrow or peripheral blood samples (Nygaard, Oslo, Norway). Isolated cell samples were then immediately cryopreserved. Total RNAs were extracted from 10^7 ^thawed cells using RNEasy^® ^Mini Kits (Qiagen Incorporation, Valencia, USA). Total RNA quantification was performed using the Nanodrop ND-1000 spectrophotometer (Thermo Fisher Scientific Incorporation, Waltham, USA) according to manufacturer recommendations. Integrity of the extracted RNAs was assessed with the Bioanalyzer 2100 and the RNA6000 Nano kit (Agilent Technologies Incorporation, Santa Clara, USA). A RNA integrity number (RIN) greater or equal to 7.00 was achieved for 203 samples (RNA degradation observed for 3 AML samples). No sign of DNA contamination was detected in any of the 206 samples analyzed. The starting amount of total RNA used for the reactions was 200 nanograms per sample, for all samples. The Illumina Total Prep RNA Amplification Kit (Applied Biosystems/Ambion, Austin, USA) was used to generate biotinylated, amplified cRNA according to the manufacturer recommendations (see additional file 1 for details). To study the leukemic blast load effect on the class prediction accuracy, besides including 22 samples with good quality control criteria that originally contained less than 60 percent of leukemic blasts, 79 artificially generated low leukemic blast load samples, via a "dilution/mixture" approach, were also assessed (see additional file 1 for details for the generation of low leukemic blast load AML samples by a "dilution/mixture" approach). Hybridization on Illumina HumanHT-12 v3 Expression BeadChips, staining and detection of cRNAs on microarrays using an I-Scan system were performed according to Illumina's protocol (see additional file 1 for details).

### Data analyses

GenomeStudio 2010.3 software (Illumina Inc., San Diego, USA) and its Gene Expression Analysis Module (version 1.8.0) were used for signal extraction and normalization (for the Training Set only). Briefly, the Invariant Rank normalization method was applied to the primary probe data obtained from the Training Set samples. Processed probe data were then filtered according to the following criteria: minimal signal intensity fold change of 1.50 across all samples; minimal probe signal intensity absolute change of 150 across all samples (choice based on the maximum expression levels of *XIST *transcript in males, and *CYorf15B *transcript in females); and maximal value for probe signal intensity of 50 000 across all samples. Among the 48 803 probes assessed on HumanHT-12 v3 Expression BeadChips, 11 779 satisfied these low stringency filtering criteria. Filtered data were then log-transformed and exported to appropriate softwares for the analyses. Regarding the Test Set samples processing, only data concerning the markers identified during the first analysis, restricted to the Training Set, were used. These raw data were log-transformed and merged to the one of the Training Set for class prediction analyses. The Class Prediction Module of ArrayMiner 5.3.3 (Optimal Design, Brussels, Belgium - http://www.optimaldesign.org), which uses a proprietary method based on grouping genetic algorithms, was used for all class prediction analyses (see additional file 1 for details) [24]. This module allowed calculating the confidence level of the class prediction for each sample, or for a given group of samples, and the fitness of the overall model. This estimator enabled optimizing the number of probes/markers to be used per class. Briefly, the confidence level of the class prediction analysis reflected the strength with which the probe/marker landed each sample into its predicted class. More precisely, for each sample, the following happened: (1) each probe/marker casted its vote for the sample being in each of the train classes, (2) the votes were summed up over all probes/markers, yielding a vote for each of the classes, (3) the score of the winner class (call it S1) was compared to the second best (call it S2), and the confidence level of the classification of the sample was computed as (S1-S2)/(S1+S2). In the Cross-validation analysis, the same was performed, except that the probe/marker set was recomputed each time, leaving out the sample being tested in order to yield an honest estimate of the worth of the method. The fitness of a class prediction model was computed on the test samples only, in order to estimate how good the class prediction (of the "unclassified" samples) was, and reflected the success with which the model was able to correctly (re)classify the already "classified" samples. The fitness of the model was in fact the result of a cross-validation (as described above) on the Training samples, with the integral part being equal to the number of correct (re)classifications, and the fractional part being computed as follows: (1) if all samples were correctly classified, the average confidence level (as defined above) of all samples, (2) if some samples were misclassified, the average confidence level of the correctly classified samples minus the average confidence level of the misclassified ones. If all samples were misclassified, the fitness of the model was zero. Omics Explorer 2.2 software (Qlucore, Lund, Sweden - http://www.qlucore.com) was used for principal-component analyses. S-Plus^® ^8.0 Enterprise Developer software (Insightful Corporation, Seattle, USA) was used for all other statistical analyses. The two datasets (Training Set and Test Set) discussed in this publication have been deposited in NCBI's Gene Expression Omnibus and are accessible through GEO Series accession number GSE34823 http://www.ncbi.nlm.nih.gov/geo/query/acc.cgi?acc=GSE34823. The dataset associated with the Training Set is also linked to the SubSeries accession number GSE34577. The dataset associated with the Test Set is also linked to the SubSeries accession number GSE34714.

## Results

### Identification of classifiers from optimal APL, t(8;21)-AML, inv(16)-AML and NK-AML samples

**Figure 1:**
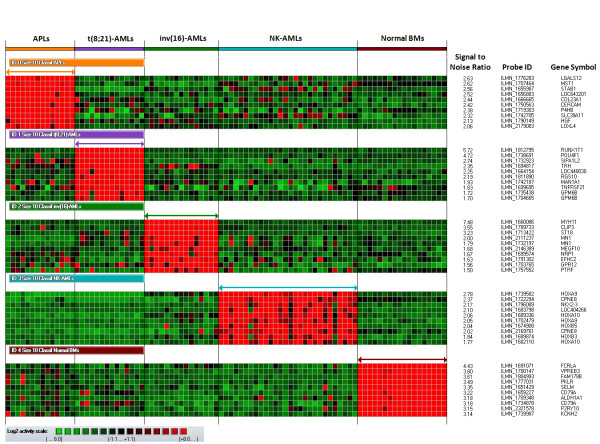
**Heatmap of the 40 markers used to define the four AML classifiers allowing the assignment of all Training Set AML samples to the correct class**. From the top to the bottom: APL, t(8;21)-AML, inv(16)-AML, and NK-AML classes - 10 markers per class (normal bone marrow class is not represented). Each column represents a sample; each row represents a marker (gene transcript). The log2 relative gene expression scale is depicted on the bottom left.

First, using the Class Prediction Module of ArrayMiner 5.3.3 software, classifiers associated with the APL, t(8;21)-AML, inv(16)-AML, NK-AML classes were defined based on 71 samples containing at least 60% of leukemic blasts and characterized by good quality control criteria (Optimal samples - Training Set). At this stage, unique and pooled normal bone marrow samples were also included in the model as the objective was to enhance the predictive capacity of the classifiers, especially for Test Set samples containing a majority of residual normal cells and a low leukemic blast load. Evaluating classifiers which included from 1 to 100 markers per class, all Training Set samples were assigned to the correct class when selecting from 3 to 46 markers per class (class prediction accuracy, sensitivity, specificity, negative and positive predictive values, 100% for each class - error rate, 0%), whether the source of leukemic cells was bone marrow or peripheral blood. The best model fitness was obtained with 8, 9 or 10 markers per classifier. To select the optimal model among these three, a principal-component analysis was performed. The highest cumulative percent of variance accounted for by the first three components was obtained with the 10-marker classifiers (cumulative percent of variance with classifiers including 10 markers, 79 percent - additional file 1, Figure S1), which led to select these for subsequent analyses (Figure 1). The best median confidence levels for the correct assignment of the Training Set samples were achieved with 10, 2, 1, and 9 markers for APLs, t(8;21)-AMLs, inv(16)-AMLs, and NK-AMLs, respectively (additional file 1, Figure S2). Regarding NK-AMLs and in agreement with previous reports, the highest median confidence levels were observed for NK-AMLs with mutated *NPM1 *and the lowest ones for NK-AMLs with neither *NPM1 *nor *FLT3 *mutations (additional file 1, Figure S3) [22-26].

### Class prediction analysis for suboptimal AML samples - Impact of the leukemic blast load

**Figure 2:**
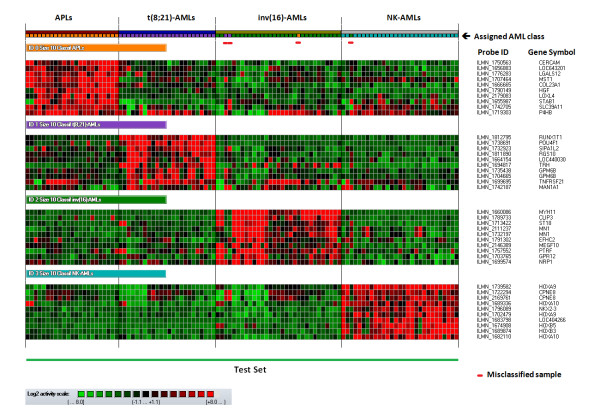
**Results of the class assignment for the 107 AML Test Set samples fulfilling all quality control criteria based on the 10-marker classifiers characterizing the APL, t(8;21)-AML, inv(16)-AML and NK-AML classes (all three AML cell lines run in duplicates included)**. Each column represents a sample; each row represents a marker (gene transcript). The log2 relative gene expression scale is depicted on the bottom left.

**Figure 3:**
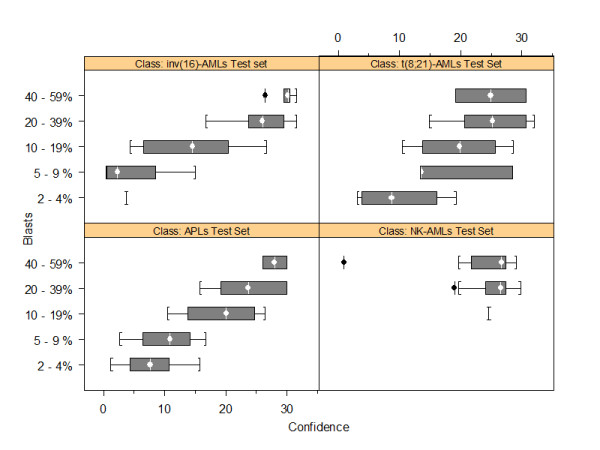
**Box plots of the confidence levels for the class assignment of the APL, t(8;21)-AML, inv(16)-AML and NK-AML Test Set samples according to their leukemic blast load**. The white vertical line and circle in the interior of the dark gray box is located at the median of the data. The width of the box is equal to the interquartile distance, which is the difference between the third and first quartiles of the data. The interquartile distance indicates the spread of the distribution for the data. The whiskers (the lines extending from the left and right parts of the box) go to the nearest value not beyond the span from the quartiles, i.e., 1.5 times the interquartile distance from the center of the data. Points beyond the whiskers are considered outliers and are drawn individually, indicated in black (+).

Using the 10-marker classifiers, the GGA-based supervised method was subsequently applied to a series of 101 Test Set samples, with optimal quality control criteria, for which the leukemic blast load was originally below 60 percent or had been artificially lowered to less than 60 percent blasts by dilution series. Only the classifiers associated with one of the four AML classes were considered for this analysis. All APL and t(8;21)-AML Test Set samples were correctly classified, even when containing as low as 2 percent blasts (Table 3 - Figure 2). Twenty six of the 29 inv(16)-AML samples were assigned to the expected class, including the 95 percent-diluted ones which contained as low as 3 percent blasts (error rate, 10 percent). In all three inv(16)-AML Test Set samples that were incorrectly classified, *CBFB*-*MYH11 *fusion gene was detected by FISH assay. For APL, t(8;21)-AML and inv(16)-AML classes, the blast load significantly influenced the confidence level of the class assignment (Figures 3 - additional file 1, Figure S4). For NK-AMLs, all but 1 of the 29 Test Set samples were correctly classified (error rate, 3 percent) (Table 3). This misclassified sample, obtained at diagnosis from UPN60, had no *NPM1, FLT3 *or *CEBPA *mutations. Its class assignment, characterized by a low confidence level (given its blast load - Table 1, additional file 1, Figure S4), was not confirmed as no PML/RARA fusion gene was detected by FISH analysis and RT-PCR assay. Across the range of leukemic blast loads studied in the Test Set, the confidence level associated with the class assignment of diluted AML samples was not different from the one achieved for undiluted low leukemic blast load samples (Table 3 - additional file 1, Figure S5). For those 101 AML Test set samples with low leukemic blast load and optimal quality control criteria, the overall error rate was 3.9 percent.

### Class prediction analysis for suboptimal AML samples - Impact of quality control criteria

When the performance of the 10-marker classifiers was assessed on 10 samples that did not fulfill all quality control criteria, all suboptimal samples were assigned to the expected class, even though their leukemic blast content was as low as 9 percent (median, 55 percent; range 9 to 100 percent) (Table 3 - additional file 1, Figure S6). The confidence level for the class assignment of these samples was not different from the one achieved for the other Test Set samples (Data not shown).

Overall, the error rate was 3.6 percent for the entire Test Set (excluding AML cell lines that were considered as controls), and for each class, sensitivity and specificity, negative and positive predictive values of the class assignments ranged from 91 to 100 percent (additional file 1, Figure S7).

## Discussion

The integration of microarray-derived data to the current workflow dealing with the prognostic evaluation of AML patients requires the technology to deliver informative data for the majority of samples, including those with suboptimal characteristics. The current study focused on this kind of samples - either with a low leukemic blast load, poor quality control criteria or both - and the ability to identify favorable-risk karyotype AMLs in such situations, using microarray-based GEP and a class prediction method based on GGA. Apart from the study of de Ridder et al, which addressed the impact of random, fixed and group-specific impurities on the results of differential gene expression analyses, using a limited set of samples and computer simulations, the influence of the leukemic blast load on class prediction accuracy based on GEP data has never been specifically studied so far [25]. To evaluate the capacity of microarray-derived predictors to correctly assigned samples with a low leukemic blast load, besides using "real" AML samples with low blast percentage, i.e., less than 60 percent, a "dilution/mixture" approach was considered [26,27]. This strategy has already been successfully applied to assess the efficiency of various normalization methods applied to microarray datasets. It consisted in diluting labeled cRNAs of originally high blast load AML samples within pools of normal bone marrow labeled cRNAs. This approach was also considered as in some AML subtypes, such as t(8;21)-AMLs, more differentiated cells (not counted as blasts) are frequently of leukemic origin, leading to under-estimate the leukemic load of some "low blast content" samples. Consequently, it became possible to accurately control the percentage of leukemic blasts within the Test Set sample down to 2 percent.

Overall, considering the 111 Test Set samples with low leukemic blast load and/or suboptimal quality control criteria, whether being peripheral blood or bone marrow samples, the predictive capacity of the GGA-derived model with 10-marker classifiers was encouraging, with specificities ranging from 0.98 to 1.00, and sensitivities ranging from 0.91 to 1.00. Moreover, considering AML samples containing 20 to 56 percent blasts, the overall error rate was lower than 1.4 percent (1 misclassified NK-AML sample out of 72). Finally, APL, t(8;21)-AML, and inv(16)-AML samples containing as low as 2 percent blasts could be correctly classified, whether the classifiers mainly relied on a single marker, such as for inv(16)-AMLs and t(8;21)-AMLs, or on the overall set of 10 markers defining the classifier, as for APL samples. However, regarding APL, t(8;21)-AML and inv(16)-AML samples, the confidence level of the class assignment was correlated to the percentage of leukemic blasts within the studied samples. For NK-AML samples, such a finding was not observed, probably because NK-AMLs represent a more heterogeneous group of leukemias as compared to t(8;21)-AMLs, inv(16)-AMLs or APLs in term of oncogenic processes. This heterogeneity likely led to a higher variance of gene expression profiles within the NK-AML class, to the detriment of the interclass variance, hence influencing the class prediction confidence level more than the leukemic blast load. This hypothesis was supported by the fact that, within the NK-AML class, the best predictive capacity of the GGA was achieved for *NPM1*-mutated samples, in accordance with previous studies, and the worst one for samples with no *NPM1 *and *FLT3 *mutations [28-31].

Unexpectedly, the worst results of the class prediction analysis were achieved for inv(16)-AML Test set samples (Sensitivity, 0.91; negative predictive value, 0.96). Among the three misclassified inv(16)-AML samples and for which the presence of *CBFB/MYH11 *fusion gene was confirmed by FISH analysis, all contained less than 20 percent blasts and two were obtained at the time of relapse - from patients UPN7 and UPN37 -. Regarding these two samples, which respectively contained 5 and 14 percent blasts, they were characterized by a low expression level of most markers defining the inv(16)-AML specific signature; especially *MYH11*, which expression level was within the estimated background signal range. It is noteworthy that for one of those two patients, UPN37, the bone marrow sample obtained at diagnosis was correctly classified, even when diluted at 50 and 75 percent (blast content, 20 and 10 percent, respectively). For this patient, a clonal evolution of the disease was suspected as CD2, CD13, CD34 and CD117 expression values, assessed by flow cytometry on gated leukemic cells, and *MYH11 *expression level (microarray data) were significantly lower in the relapse sample as compare to the ones observed in the diagnostic sample. Verhaak et al in their study using Affymetrix GeneChips reported that *MYH11 *expression level was sufficient to identify inv(16)-AMLs [10]. The present results suggest that the use of *MYH11 *as the sole marker for the inv(16)-AML class could lead to unstable results in a class prediction model, especially in low leukemic blast load samples or in case of a clonal evolution at relapse. Of note, the inv(16)-AML signature developed on the AMLProfiler™ kit http://www.skyline-diagnostics.com, which uses the Affymetrix™ platform, also includes a set of 19 markers [32].

As reported by Kohlmann et al., sub-optimal AML samples with poor quality control criteria, mainly because of low amounts of hybridized cRNAs, were all correctly classified, even though half of these contained less than 60 percent blasts (down to 9 percent for UPN90) [33]. Studying the impact of RNA degradation on GEP analyses, similar results have been recently reported by Opitz et al on a different microarray platform [34]. As in the present study, they showed that useful information could still be obtained from thermically degraded RNA; at least to a certain extent, and depending on the length of the mRNA molecules. Interestingly, even AML samples for which a third of the required cRNA amount was hybridized on BeadChips could be assigned to the correct class in the present study, suggesting that the biological variance between the AML classes was higher than the one related to technical variability or artifacts. This result could also be related to the fact that, during the sample processing, the TotalPrep RNA Amplification kit that was used, did not required any fragmentation step, which could further alter poor quality RNA or lower the already reduced cRNA amounts. These encouraging results, on a limited set of poor quality samples, could also be related to the classification method used, as the GGA-based class prediction strategy aimed at finding the most influential markers for each AML class (top markers with the highest inter-class variance and the lowest intra-class variance). This GGA-based class prediction method was indeed associated with the lowest error rate and the highest predictive accuracy when compared to other supervised methods, such as support vector machines, random forests, artificial neural networks, k-Nearest Neighbor or nearest shrunken centroids (also known as PAM) (data not shown).

Although, in most cases, these favorable prognostic chromosomal abnormalities and/or their related gene products can be detected by karyotype, FISH or PCR assay, they represent main targets for a microarray-based class prediction analysis to be identified before considering GEP as a useful tool in a routine workflow for prognostic assessment of AML patients. The fact that the AMLProfiler, a microarray-derived kit based on the Affymetrix™ platform, has been recently commercialized with the aim of achieving a molecular diagnosis for APLs, t(8;21)-AMLs and inv(16)-AMLs, respectively using 27, 31 and 19 markers, emphasizes this assumption [32].

Finally, regarding the classifiers associated with APLs, t(8;21)-AMLs or inv(16)-AMLs, 18 of the 28 markers identified in this study (64 percent) had already been reported in previous studies using various microarray technologies (additional file 1, Table S1). These findings (1) confirm that the Illumina bead-based technology is as reliable, robust and sensitive as other microarray technologies developed by commercial manufacturers or academic facilities, and (2) suggest that the GGA-derived class prediction approach is a highly efficient one as it required a limited set of ten markers per class to achieve accurate class assignments. Furthermore, this study has identified new AML markers that will need further studies to delineate their role in the leukemogenic events involved in APLs (*CERCAM, COL23A1*, LOC643201, *LOXL4, SLC39A11*), t(8;21)-AMLs (LOC440030, *TNFRSF21*), and inv(16)-AMLs (*EFHC2, GPR12, MEGF10*).

## Conclusions

In more than 96 percent of the suboptimal cases, using a microarray-derived GEP approach and a GGA-based class prediction method, favorable cytogenetic risk AML samples with low leukemic blast load and/or poor quality control criteria could be correctly assign to the appropriate class with a limited set of markers, allowing to consider GEP as a useful tool in a routine workflow for prognostic assessment of AML patients.

## List of abbreviations used

APL: acute promyelocytic leukemia; AML: acute myeloid leukemia; GEP: gene expression profiling; GGA: grouping genetic algorithms; NK-AML: cytogenetically normal acute myeloid leukemia; RIN: RNA integrity number.

## Competing interests

The authors declare that they have no competing interests.

## Authors' contributions

PhG was the principal investigator and takes primary responsibility for the paper. DRB, OB and PhG contribute to the conception of design. DRB, PCL, AC, LB, FG, IL, MHB, AB, AST, MZ, YD, NI, OB, PhG contribute to the collection and assembly of data. DRB, OB, YD, MZ, NI, PhG contribute to the data analysis and interpretation. DRB, PhG contribute to manuscript writing. DRB, OB, PCL, AC, LB, FG, IL, MHB, AB, AST, MZ, YD, NI, PhG contribute to the drafting of the article, revise it and approved the final version. PhG coordinated the research.

## Authors' information

PhG is the Head of Plateforme SNP, Transcriptome & Epigénomique, i.e., the genomic platform of Angers University Hospital, and is also an Associate Editor at BMC Genomics and a member of the American Society of Human Genetics.

## Pre-publication history

The pre-publication history for this paper can be accessed here:

http://www.biomedcentral.com/1755-8794/5/6/prepub

## Supplementary Material

Additional file 1 **Sample preparation for gene expression profiling**. - Generation of biotinylated, amplified cRNA using The Illumina Total Prep RNA Amplification Kit (Applied Biosystems/Ambion, Austin, USA). - Generation of low leukemic blast load AML samples by a "dilution/mixture" approach. - Hybridization on Illumina HumanHT-12 v3 Expression BeadChips, staining and detection of cRNAs on microarrays using an I-Scan system. Additional table S1 - Citations in previous studies of the identified markers. Additional figures S1 to S7. Figure S1. Three-dimensional projection of the 3 principal components in a principal-components analysis of APL, t(8;21)-AML, inv(16)-AML, NK-AML, and Normal Bone Marrow samples belonging to the Training Set, with the use of the 10-marker classifiers. Figure S2. Per class median confidence level of the assignment for APL, t(8;21)-AML, inv(16)-AML, NK-AML and normal bone marrow samples belonging to the Training Set according to the number of markers per class (from 1 to 100 markers per class). Figure S3. Median confidence level of the class assignments for the NK-AML samples belonging to the Training Set according to *FLT3 *and *NPM1 *mutational status and the number of markers per class (from 1 to 100 markers per class). Figure S4. Relationship between the percentage of leukemic blasts within the 101 Test Set samples (X axis) and the confidence level of their class assignments (Y axis) (samples with poor quality control criteria and AML cell lines were excluded). Figure S5. Confidence level of the class assignments according to the percentage of leukemic blasts including comparisons between diluted and not diluted samples. Figure S6. Results of the class assignment for the 10 AML Test Set samples with suboptimal quality control criteria based on the 10-marker classifiers characterizing the APL, t(8;21)-AML, inv(16)-AML and NK-AML classes. Figure S7. Sensitivity, specificity, negative and positive predictive values of the prediction model for the class assignment of the 111 Test Set samples (all AML samples with or without optimal quality control criteria - AML cell line samples excluded) to the APL, t(8;21)-AML, inv(16)-AML and NK-AML classes with 10-marker classifiers. Click here for file
