# Supplementary material for: Routine use of microarray-based gene expression profiling to identify patients with low cytogenetic risk acute myeloid leukemia: accurate results can be obtained even with suboptimal samples
Source: BMC Med Genomics. 2012 Jan 30;5:6. doi: 10.1186/1755-8794-5-6 (PMC3284426; doi:10.1186/1755-8794-5-6)
Supplement: Additional file 1 — Sample preparation for gene expression profiling. - Generation of biotinylated, amplified cRNA using The Illumina Total Prep RNA Amplification Kit (Applied Biosystems/Ambion, Austin, USA). - Generation of low leukemic blast load AML samples by a "dilution/mixture" approach. - Hybridization on Illumina HumanHT-12 v3 Expression BeadChips, staining and detection of cRNAs on microarrays using an I-Scan system. Additional table S1 - Citations in previous studies of the identified markers. Additional figures S1 to S7. Figure S1. Three-dimensional projection of the 3 principal components in a principal-components analysis of APL, t(8;21)-AML, inv(16)-AML, NK-AML, and Normal Bone Marrow samples belonging to the Training Set, with the use of the 10-marker classifiers. Figure S2. Per class median confidence level of the assignment for APL, t(8;21)-AML, inv(16)-AML, NK-AML and normal bone marrow samples belonging to the Training Set according to the number of markers per class (from 1 to 100 markers per class). Figure S3. Median confidence level of the class assignments for the NK-AML samples belonging to the Training Set according to FLT3 and NPM1 mutational status and the number of markers per class (from 1 to 100 markers per class). Figure S4. Relationship between the percentage of leukemic blasts within the 101 Test Set samples (X axis) and the confidence level of their class assignments (Y axis) (samples with poor quality control criteria and AML cell lines were excluded). Figure S5. Confidence level of the class assignments according to the percentage of leukemic blasts including comparisons between diluted and not diluted samples. Figure S6. Results of the class assignment for the 10 AML Test Set samples with suboptimal quality control criteria based on the 10-marker classifiers characterizing the APL, t(8;21)-AML, inv(16)-AML and NK-AML classes. Figure S7. Sensitivity, specificity, negative and positive predictive values of the prediction model for the class assignment [file 1755-8794-5-6-S1.DOC]

**Design and Methods – Additional information**

***Sample preparation for gene expression profiling***

**Generation of biotinylated, amplified cRNA using The Illumina Total Prep RNA Amplification Kit (Applied Biosystems / Ambion, Austin, USA).**

Briefly, total RNAs were reverse transcribed in a reaction volume of 20 microliters comprising 1xFirst Strand Buffer, T7-Oligo(dT) primers, dNTP mix, RNase inhibitor, and ArrayScript enzyme. After a 2-hour incubation at 42°C, 80 microliters of Second Strand Master Mix, including 1x Second Strand Buffer, dNTP mix, DNA polymerase and RNase H, were added to each sample and incubated at 16°C for an additional 2 hours. The resulting cDNAs were then purified by adding 250 microliters of cDNA Binding Buffer to each sample, and passing the mixture through a cDNA Filter Cartridge by centrifugation at 10,000xg for one minute. Filters were then washed with Wash Buffer (500 microliters), and dried by centrifugation for an additional minute. cDNAs were eluted with a total of 19 microliters of Nuclease-free Water at 55°C. In Vitro Transcription Master Mix (7,5 microliters), comprising 1x T7 Reaction Buffer, T7 enzyme mix and Biotin-NTP mix, was added to each cDNA sample and incubated at 37°C for 14 hours. The reaction was stopped by adding 75 microliters of Nuclease-free Water. The resulting biotin-labeled cRNAs were then purified by adding 350 microliters of cRNA Binding Buffer and 250 microliters of 100% ethanol to each sample before passing through a cRNA filter cartridge under centrifugation at 10,000xg for one minute. Filters were then washed with 650 microliters of Wash Buffer, and dried by centrifugation for an additional minute. cRNAs were then eluted with 100 microliters of Nuclease-free water preheated to 55°C, and quantified using the Nanodrop ND-1000 (NanoDrop, Wilmington, DE, USA).

**Generation of low leukemic blast load AML samples by a “dilution/mixture” approach.**

For this group of samples, cRNA of AML samples containing 40 to 100 percent blasts (median, 80 percent) were diluted at 50, 75, 90 and 95 percent within one of the five normal marrow pools (random assignment of the AML samples to be diluted into one of the five normal bone marrow pools). Briefly, before being mixed together, the concentration of each sample was adjusted to 150 nanograms per microliter. Five normal pools of cRNA used for the dilutions were obtained by mixing an equal volume of cRNA amplified from normal bone marrow cells obtained from 12 healthy donors (Pool1 = Donors 1 + 2 + 3 + 4 + 5 + 6 + 7 + 8 + 9; Pool2 = Donors 1 + 2 + 3 + 4 + 7; Pool3 = Donors 1 + 2 + 3 + 4 + 7 + 10 + 11 + 12; Pool 4 = Donors 4 + 7 + 10 + 11 + 12; pool 5 = Donors 5 + 7 + 10 + 11 + 12). In order to obtain a dilution of the AML sample cRNAs at 50 percent, cRNA from each AML patient was mixed with an equal volume of the cRNA from one of the five normal pools. A part of this dilution mix was diluted again either with an equal volume of cRNA from one of the five normal pools to get a dilution at 75 percent, or five times to obtain a blast load diluted at 90 percent. Finally, samples diluted at 95 percent were obtained by mixing an equal volume of cRNA diluted at 90 percent with cRNA from one of the five normal pools. Therefore, AML samples diluted at 50 percent (n=28) and 75 percent (n=27) contained 20 to 50 percent (median, 40 percent) and 10 to 25 percent (median, 20 percent) blasts, respectively. Acute myeloid leukemia samples diluted at 90 percent (n=12) and 95 percent (n=12) contained 4 to 10 percent and 2 to 5 percent blasts, respectively.

**Hybridization on Illumina HumanHT-12 v3 Expression BeadChips, staining and detection of cRNAs on microarrays using an I-Scan system.**

Briefly, except in 8 cases for which the obtained cRNA amount was low, ranging from 260 to 715 nanograms (APL, n=1; t(8;21)-AML, n=1; inv(16)-AML, n=6), 750 nanograms of each cRNA adjusted to a volume of 5 microliters were mixed to 10 microliters of HYB mix pre-warmed in a 58°C oven for 10 minutes. After 5 minutes incubation at 65°C, 15 microliters of each assay sample were loaded onto the center of each array’s inlet port. Slides were then placed in a BeadChip Hyb Chamber and incubated in a 58°C oven for 18 hours with rotation. After hybridization, each BeadChip was submerged in Wash E1BC solution to remove their cover seal and transferred into the Hybex Waterbath insert containing Hight-Temp Wash buffer to be incubated 10 minutes at 55°C. Slides were then washed, successively in Wash E1BC solution for 5 minutes, 100% ethanol for 10 minutes and again in Wash E1BC solution for 2 minutes, on an orbital shaker at 120 rpm. In order to block and detect BeadChips, slides were incubated 10 minutes with Block E1 buffer, followed by 10 minutes incubation with Block E1 buffer containing 1microgram per milliliter of streptavidin-Cy3 (Amersham, GE Healthcare, Chalfont St Giles, UK). These incubations were performed on a rocker platform with rocker shaking at 50 rpm. After a last wash in Wash E1BC for 5 minutes with orbital shake at 120 rpm, slides were dried by centrifugation at 275xg for 4 minutes at 25°C. BeadChips were then stored in the dark until they were scanned using the I-Scan reader (Illumina Inc., San Diego, USA).

The HumanHT-12 v3 Expression BeadChip assesses a total of 48 803 marker probes, of which 27 455 are NM coding transcripts; and 7 870 are XM coding transcripts (RefSeq Content, Build 36.2, Release 22). It also contains 12 837 experimentally confirmed mRNA sequences that aligned to EST clusters (UniGene, Build 199).

**Additional table S1 – Citations in previous studies of the identified markers**

**APL classifier**

CERCAM new marker

COL23A1 new marker

HGF 1, 3, 4, 5, 8

LGALS12 5, 8

LOC643210 new marker

LOXL4 new marker

MST1 1, 4, 8

P4HB 1

SLC39A11 new marker

STAB1 4, 6, 8

**t(8;21)-AML classifier**

GPM6B 5, 7

LOC440030 new marker

MAN1A1 5

POU4F1 1, 2, 4, 5, 6, 7, 8

RGS10 2, 6

RUNX1T1 1, 2, 4, 5, 6, 7, 8

SIPA1L2 5, 8

TNFRSF21 new marker

TRH 1, 5, 6, 7, 8

**inv(16)-AML classifier**

CLIP3 1, 3, 7, 8

EFHC2 new marker

GPR12 new marker

MEGF10 new marker

MN1 1, 4, 7, 8

MYH11 1,2, 4, 5, 6, 7, 8

NRP1 1, 4

PTRF new marker

ST18 1, 4

1. Valk PJ, Verhaak RG, Beijen MA, et al. Prognostically useful gene-expression profiles in acute myeloid leukemia. N Engl J Med 2004,350:1617-28.

2. Schoch C, Kohlmann A, Schnittger S, et al. Acute myeloid leukemias with reciprocal rearrangements can be distinguished by specific gene expression profiles. Proc Natl Acad Sci USA 2002,99:10008-13.

3. Gutiérrez NC, López-Pérez R, Hernández JM, et al. Gene expression profile reveals deregulation of genes with relevant functions in the different subclasses of acute myeloid leukemia. Leukemia 2005,19:402-9.

4. Ross ME, Mahfouz R, Onciu M, et al. Gene expression profiling of pediatric acute myelogenous leukemia. Blood 2004,104:3679-87.

5. Verhaak RG, Wouters BJ, Erpelinck CA, et al. Prediction of molecular subtypes in acute myeloid leukemia based on gene expression profiling. Haematologica 2009,94:131-4.

6. Kohlmann A, Schoch C, Schnittger S, et al. Molecular characterization of acute leukemias by use of microarray technology. Genes Chromosomes Cancer 2003,37:396-405.

7. Ichikawa H, Tanabe K, Mizushima H, et al. Common gene expression signatures in t(8;21)- and inv(16)-acute myeloid leukaemia. Br J Haematol 2006,135:336-47.

8. Balgobind BV, Van den Heuvel-Eibrink MM, De Menezes RX, et al. Evaluation of gene expression signatures predictive of cytogenetic and molecular subtypes of pediatric acute myeloid leukemia. Haematologica 2011,96:221-30.

**Additional figures S1 to S7**

**
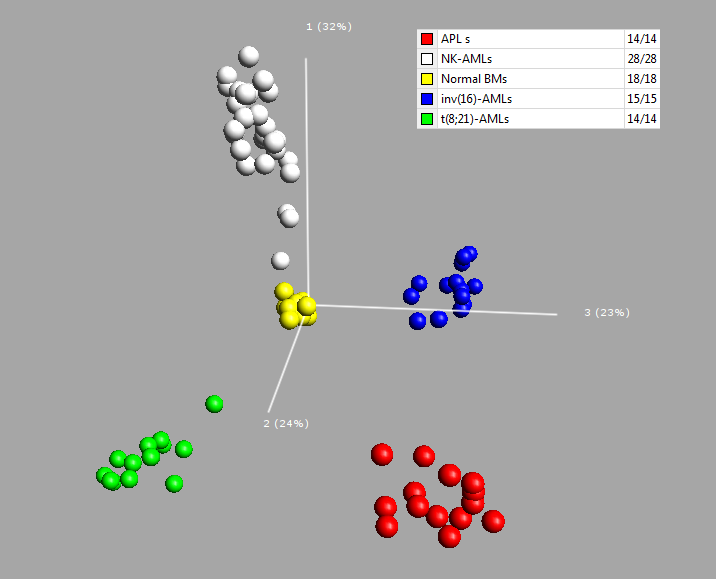
**

**Figure S1.** Three-dimensional projection of the 3 principal components in a principal-components analysis of APL, t(8;21)-AML, inv(16)-AML, NK-AML, and Normal Bone Marrow samples belonging to the Training Set, with the use of the 10-marker classifiers (First, second and third components represented 32%, 24%, and 23% of the variance, giving a cumulative percent of variance of 79%). Samples are color-coded as indicated.


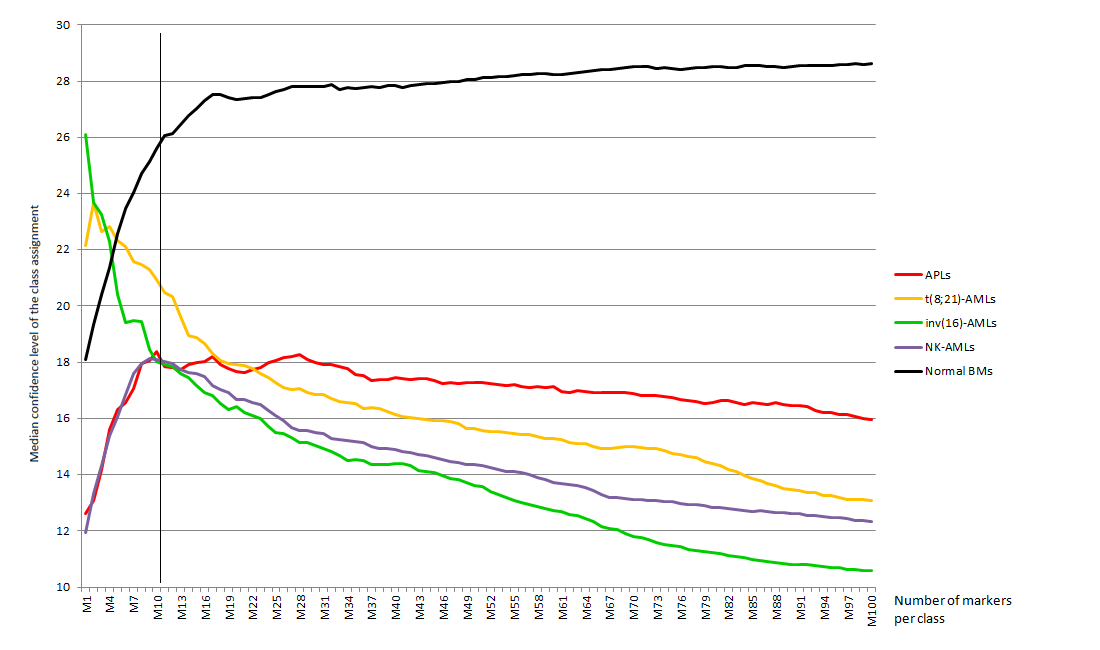


**Figure S2.** Per class median confidence level of the assignment for APL, t(8;21)-AML, inv(16)-AML, NK-AML and normal bone marrow samples belonging to the Training Set according to the number of markers per class (from 1 to 100 markers per class).

**
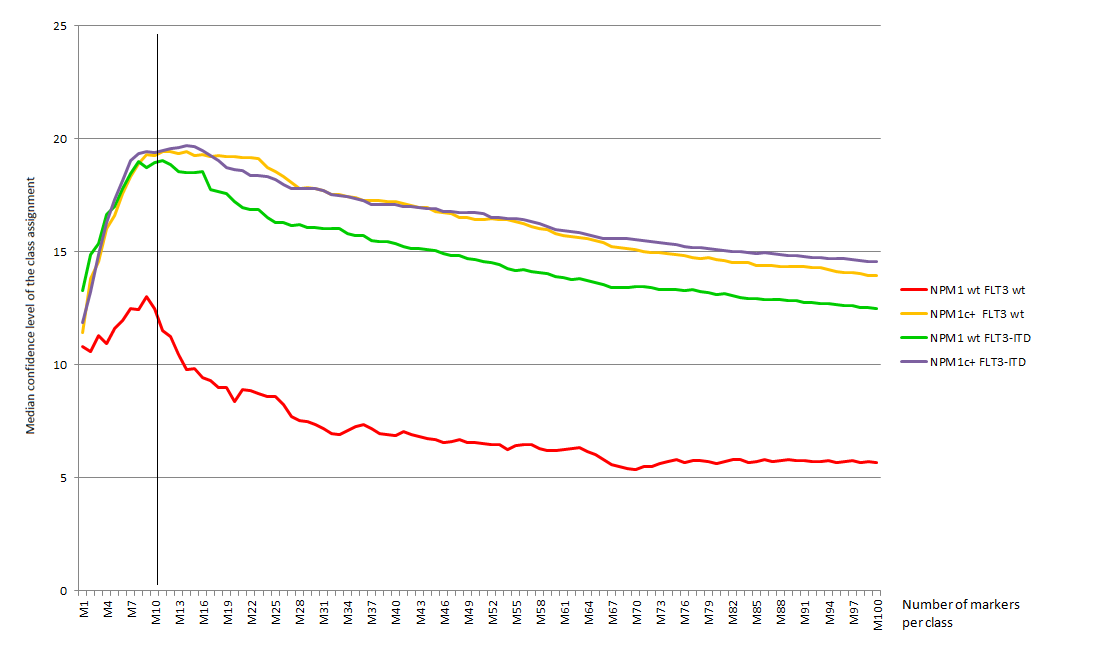
**

**Figure S3.** Median confidence level of the class assignments for the NK-AML samples belonging to the Training Set according to *FLT3* and *NPM1* mutational status and the number of markers per class (from 1 to 100 markers per class).

**
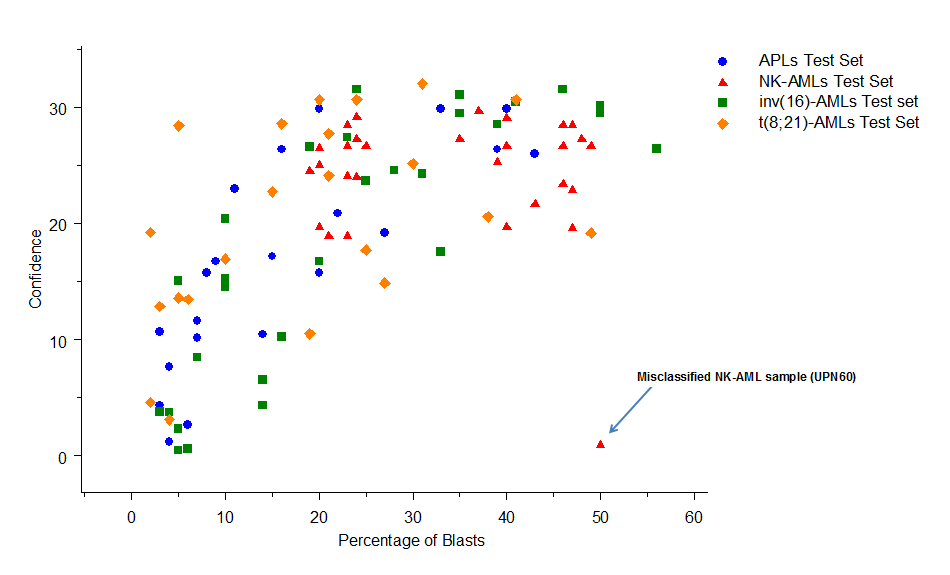
**

**Figure S4.** Relationship between the percentage of leukemic blasts within the 101 Test Set samples (X axis) and the confidence level of their class assignments (Y axis) (samples with poor quality control criteria and AML cell lines were excluded). NK-AML sample from UPN60 was assigned to the inv(16)-AML class with a confidence level of 0.95 despite containing 50 percent blasts. With UPN60 sample included, R^2^ = 0.3870, Pearson correlation coefficient 0.6221, p < 0.0001. Without UPN60 sample, R^2^ = 0.4695, Pearson correlation coefficient 0.6852, p < 0.0001.

**
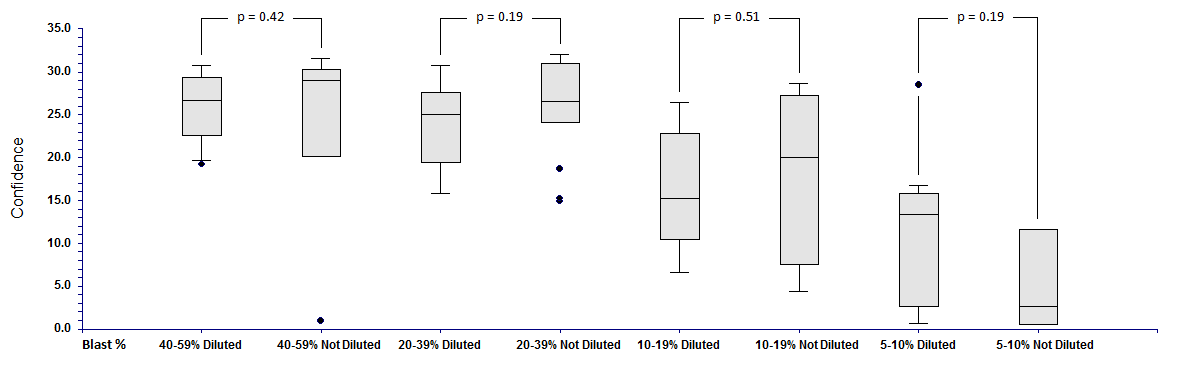
**

**Figure S5.** Confidence level of the class assignments according to the percentage of leukemic blasts including comparisons between diluted and not diluted samples. Confidence levels of the class assignments are presented using box plots. The black horizontal line in the interior of the grey box is located at the median of the data. The height of the box is equal to the interquartile distance, which is the difference between the third and first quartiles of the data. The interquartile distance indicates the spread of the distribution for the data. The whiskers (the lines extending from the bottom and the top parts of the box) go to the nearest value not beyond the span from the quartiles, i.e., 1.5 times the interquartile distance from the center of the data. Points above or below the whiskers are considered outliers and are drawn individually, indicated as black dots). p-values represent the results of Wilcoxon rank-sum test for differences in medians between diluted and not diluted groups.

**
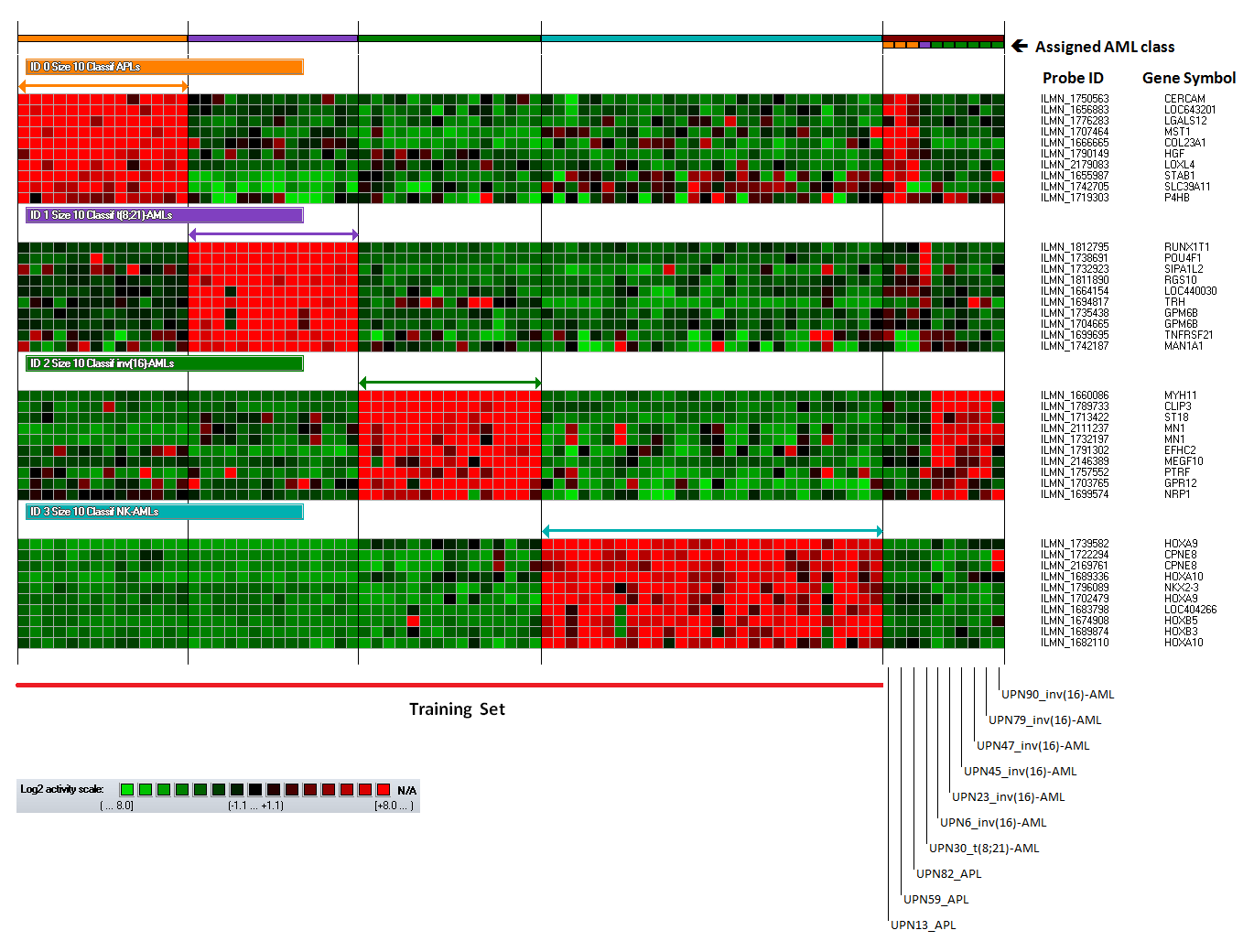
**

**Figure S6.** Results of the class assignment for the 10 AML Test Set samples with suboptimal quality control criteria based on the 10-marker classifiers characterizing the APL, t(8;21)-AML, inv(16)-AML and NK-AML classes. Each column represents a sample; each row represents a marker (gene transcript). The log2 relative gene expression scale is depicted on the bottom left.

**
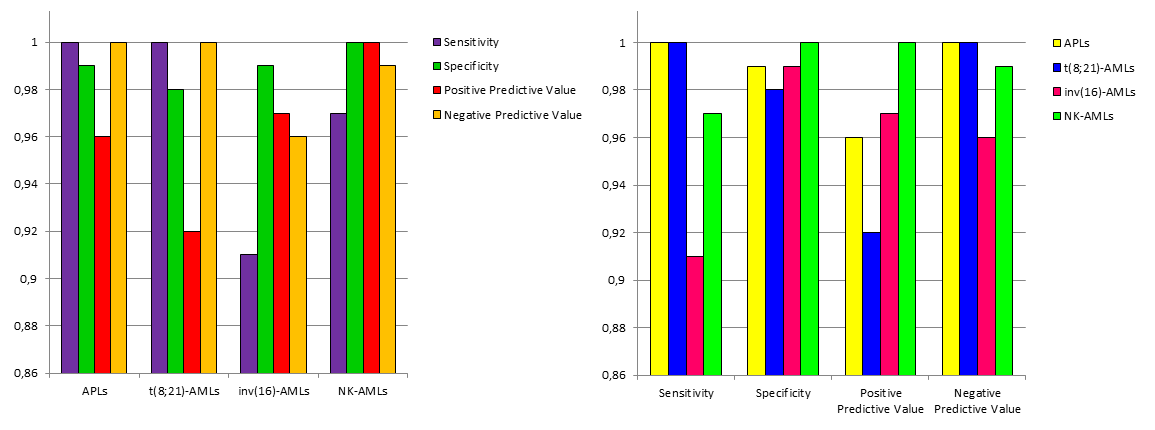
**

**Figure S7.** Sensitivity, specificity, negative and positive predictive values of the prediction model for the class assignment of the 111 Test Set samples (all AML samples with or without optimal quality control criteria – AML cell line samples excluded) to the APL, t(8;21)-AML, inv(16)-AML and NK-AML classes with 10-marker classifiers.
